# Metagenomics Revealed a New Genus ‘*Candidatus* Thiocaldithrix dubininis’ gen. nov., sp. nov. and a New Species ‘*Candidatus* Thiothrix putei’ sp. nov. in the Family *Thiotrichaceae*, Some Members of Which Have Traits of Both Na^+^- and H^+^-Motive Energetics †

**DOI:** 10.3390/ijms241814199

**Published:** 2023-09-17

**Authors:** Nikolai V. Ravin, Maria S. Muntyan, Dmitry D. Smolyakov, Tatyana S. Rudenko, Alexey V. Beletsky, Andrey V. Mardanov, Margarita Yu. Grabovich

**Affiliations:** 1 Institute of Bioengineering, Research Center of Biotechnology, Russian Academy of Sciences, Leninsky Prospect, 33-2, 119071 Moscow, Russia; nravin@biengi.ac.ru (N.V.R.); mortu@yandex.ru (A.V.B.); mardanov@biengi.ac.ru (A.V.M.); 2 Belozersky Institute of Physico-Chemical Biology, Lomonosov Moscow State University, Leninskie Gory, 119991 Moscow, Russia; 3 Department of Biochemistry and Cell Physiology, Voronezh State University, Universitetskaya pl., 1, 394018 Voronezh, Russia; songolifreya@mail.ru (D.D.S.); ipigun6292@gmail.com (T.S.R.)

**Keywords:** filamentous colorless sulfur bacteria, genome, metagenome-assembled genome, ‘*Candidatus* Thiocaldithrix dubininis’ gen. nov., sp. nov., ‘*Candidatus* Thiothrix putei’ sp. nov., H^+^-motive ATPase, Na^+^-motive ATPase, Na^+^-motive PPase

## Abstract

Two metagenome-assembled genomes (MAGs), GKL-01 and GKL-02, related to the family *Thiotrichaceae* have been assembled from the metagenome of bacterial mat obtained from a sulfide-rich thermal spring in the North Caucasus. Based on average amino acid identity (AAI) values and genome-based phylogeny, MAG GKL-01 represented a new genus within the *Thiotrichaceae* family. The GC content of the GKL-01 DNA (44%) differed significantly from that of other known members of the genus *Thiothrix* (50.1–55.6%). We proposed to assign GKL-01 to a new species and genus ‘*Candidatus* Thiocaldithrix dubininis’ gen. nov., sp. nov. GKL-01. The phylogenetic analysis and estimated distances between MAG GKL-02 and the genomes of the previously described species of the genus *Thiothrix* allowed assigning GKL-02 to a new species with the proposed name ‘*Candidatus* Thiothrix putei’ sp. nov. GKL-02 within the genus *Thiothrix*. Genome data first revealed the presence of both Na^+^-ATPases and H^+^-ATPases in several *Thiothrix* species. According to genomic analysis, bacteria GKL-01 and GKL-02 are metabolically versatile facultative aerobes capable of growing either chemolithoautotrophically or chemolithoheterotrophically in the presence of hydrogen sulfide and/or thiosulfate or chemoorganoheterotrophically.

## 1. Introduction

Before 2018, all the representatives of the filamentous colorless sulfur-oxidizing bacteria that form rosettes and accumulate elemental sulfur inclusions in their cells were united into the genus *Thiothrix* within the family *Thiotrichaceae*. In 2018, based on the sequence identity of 16S RNA and several other genes, Boden reclassified all the representatives of the genus *Thiothrix* into three families, *Thiotrichaceae*, *Thiolineaceae*, and *Thiofilaceae*, within the order *Thiotrichales*, and in addition to them included in this order another family *Leucotrichaceae* [1]. During that reclassification, two genera, *Leucothrix* and *Cocleimonas*, were assigned to the family *Leucotrichaceae*.

**Figure 1:**
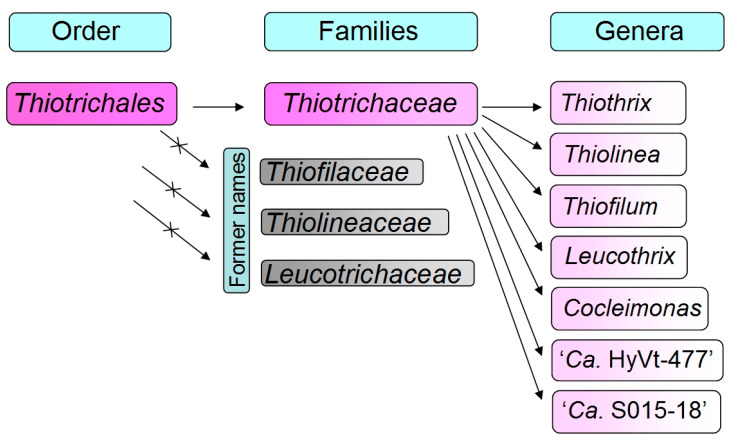
Reclassification of genera and families composing the order *Thiotrichales* according to genome taxonomic database (GTDB) (Release 08-RS214 (28 April 2023)).

Somewhat later, after the symposium at FEMS-2019, it was decided that Bergey’s Manual of Systematics of Archaea and Bacteria (BMSAB) would use the classification of the continuously updated Genome Taxonomy Database (GTDB) (https://gtdb.ecogenomic.org/, accessed on 28 June 2023) and its phylogenomic resource [2,3]. As a result, the order *Thiotrichales* (https://gtdb.ecogenomic.org/tree?r=o__Thiotrichales, accessed on 28 June 2023) includes only family *Thiotrichaceae* numbering seven genera (Figure 1), where five of them are validated genera, namely *Thiothrix*, *Thiolinea*, *Thiofilum*, *Leucothrix* and *Cocleimonas*, and two nonculturable candidate genera, HyVt-477 and S015-18 represented only by MAGs.

Representatives of the first three genera are unified by the following common phenotype: they are filamentous colorless sulfur bacteria forming rosettes and subcellular inclusion of elemental sulfur under the growth in the presence of hydrogen sulfide and thiosulfate. Until now, their taxonomy is far from perfect. On the one hand, all the representatives of this group are microorganisms that are difficult to cultivate and, therefore, difficult to isolate as pure cultures. On the other hand, as a rule, all the representatives share a common pattern of major metabolic pathways. In general, all the above makes the reliable classification of new isolates noticeably more difficult.

At this stage of the studies, molecular phylogenetics brings significant contributions to the solution of the problem, while metagenomic analysis allowed the study of the communities of filamentous colorless sulfur bacteria by obtaining genomes of new representatives. These methods compensate for the limited number of species and genera as well as genome sequences in view of the lack of pure cultures.

Despite significant advances in the molecular phylogenetic classification of filamentous colorless sulfur bacteria (FCSB), the genera of the family *Thiotrichaceae* are still heterogeneous and include genetically unrelated species and genera. With numerous examples, genetic tools have been shown to differentiate FCSB species and genera. In our previous publications, we reported obtaining pure cultures and genomes assembled from metagenomes of sulfur biotopes, presenting them as new species of the genus *Thiothrix* [4]. These studies have noticeably extended the mentioned group of bacteria during the past several years, resulting in substantial changes in the taxonomy of this group [5,6,7]. Recently, we succeeded in assembling two genomes from a metagenome of microbial fouling from a hydrogen sulfide-rich thermal spring. In the presented study, phylogenetically, we assigned one of these genomes to a new genus within the family *Thiotrichaceae* and denoted it as ‘*Candidatus* Thiocaldithrix dubininis’ gen. nov., sp. nov. GKL-01. The second genome we assigned to a new species within the genus *Thiothrix* and named ‘*Candidatus* Thiothrix putei’ sp. nov. GKL-02.

As it was written earlier, members of the order *Thiotrichales* are often inhabitants of niches with microoxic conditions [6,8,9,10,11,12]. This environment is formed due to the high content of sulfur compounds with low redox potential. Among members of the order *Thiotrichales*, many species are also able to exist in both microoxic and anaerobic conditions. It is known that many bacteria capable of living under anaerobic conditions are often provided by energy-containing macroergic phosphates via the function of the Na^+^-motive energy-transforming pumps such as Na^+^-ATPases [13,14,15,16]. In the present study, we have elucidated which of the major energy transducers of the phosphate metabolism group could provide biological energy to members of this order. In the genomes of several species of the family *Thiotrichaceae*, we were able to identify Na^+^- and H^+^-pump genes, exemplified by energy-generating ATPases and PPases. Due to the availability of metagenomic data, which revealed a large number of new species within taxa of this order, it became possible to analyze a number of conditions and characteristics that could affect such a feature.

## 2. Results and Discussion

### 2.1. Metagenome and Genome Assembly

**Figure 2:**
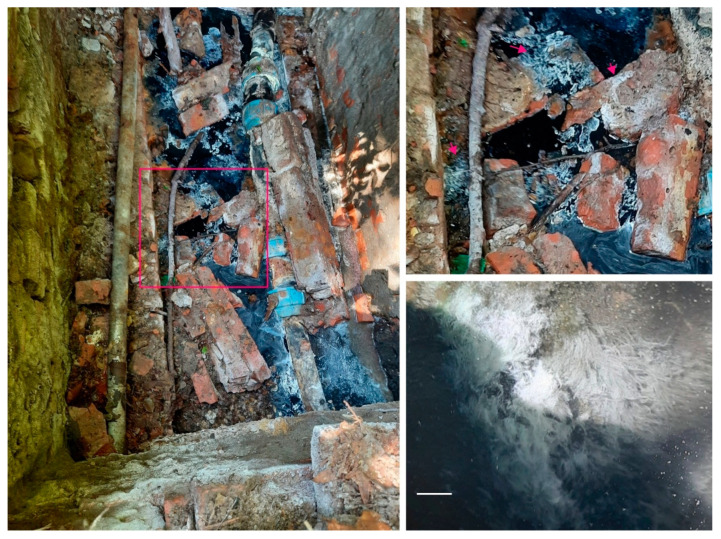
Natural fouling with a predominance of representatives of the genus *Thiothrix* in the well of a natural hydrogen sulfide-rich thermal spring: general view of the well (**left panel**). The sampling site, highlighted in a magenta rectangle (**left panel**), is shown in 2.5 × magnification (**right panel**, **top**). Fouling as white mats and rosettes on the surface of rocks and hydrogen sulfide-rich black slurry is selectively shown with magenta arrows (**right panel**, **top**). The mat from which the GKL metagenome was isolated (magenta arrow on the **left**, **right panel**, **top**) is shown in 9 × magnification (**right panel**, **bottom**). Sulfide thermal spring in the town of Goryachiy Klyuch, Krasnodar region, Russia. Bar, 1 cm (**lower left corner**, **right panel**, **bottom**).

The specimen GKL for metagenome analysis was sampled from a sulfide mat in the zone of a sulfide hydrogen thermal spring in the North Caucasus (44°38′ N, 39°08′ E), the town of Goryachiy Klyuch, Krasnodar region, Russia (Figure 2). Water characteristics in the sampling site were as follows: T 35 °C, pH 7.5–8.0, total mineralization 1.56–1.65 g/L, oxygen concentration 0.5–1 mg/L, and sulfide concentration 0.13 mM.

A total of 22,275 high-quality 16S rRNA gene sequences were determined to characterize the composition of the microbial mat at Goryachiy Klyuch spring. The microbial community was dominated by the representatives of several phyla: *Pseudomonadota* (former *Proteobacteria*, 60.8% of all 16S rRNA gene sequences including 60.1% of *Gammaproteobacteria*), *Campylobacterota* (16.2%), *Firmicutes* (5.6%), and *Bacteroidota* (4.5%). Representatives of the family *Thiotrichaceae* of the phylum *Pseudomonadota* accounted for 48.3% of the microbial mat community. Among them, two OTUs dominated with shares of 36.3% and 11.0%, which, according to the NCBI database, were identified as the representatives of the genus *Thiothrix*.

Two metagenome-assembled genomes (MAGs) were obtained from the GKL metagenome. *Thiotrichaceae* sp. GKL-01 and *Thiothrix* sp. GKL-02 MAGs were obtained as circular 3,251,546 b.p. and 4,277,058 b.p. long chromosomes, respectively. Both MAGs are 100% complete, according to CheckM2 estimates. The genome GKL-01 annotation revealed three copies of 16S-23S-5S rRNA operon, 49 tRNA genes, and 3021 genes encoding potential proteins. The GC content in the genome GKL-01 accounted for 44%. The genome GKL-02 annotation revealed three copies of 16S-23S-5S rRNA operon, 46 tRNA genes, and 4134 genes encoding potential proteins. GC content in the genome GKL-02 accounted for 50.08%. The 16S rRNA gene sequences of these two MAGs correspond to a pair of OTUs of the family *Thiotrichaceae* revealed in the microbial mat by 16S rRNA gene profiling. The main characteristics of the genomes are presented in Table 1, and further details according to the minimal standards for the description of MAGs [17] are provided in Supplementary Materials (Table S1).

### 2.2. Phylogenetic Analysis

**Figure 3:**
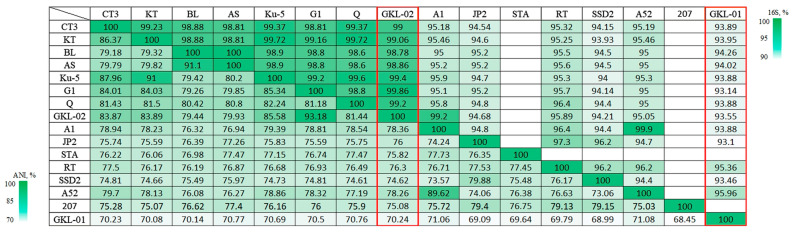
Heatmap of 16S rRNA gene sequence similarity and pairwise ANI values (%) for the assembled genomes of *Thiothrix*. *T*. *winogradskyi* CT3^T^ (GCA_021650935.1); ‘*Ca*. Thiothrix sulfatifontis’ KT (GCA_022828425.1); *T*. *lacustris* BL^T^ (GCF_000621325.1); *T*. *litoralis* AS^T^ (GCF_017901135.1); *T*. *subterranea* Ku-5^T^ (GCF_016772315.1); *T*. *caldifontis* G1^T^ (GCF_900107695.1); *Thiothrix* sp. STA_22 (GCA_028714775.1); *T*. *unzii* A1^T^ (GCA_017901175.1); *T*. *nivea* JP2^T^ (GCF_000260135.1); *T*. *fructosivorans* Q^T^ (GCA_017349355.1); *Ca*. Thiothrix moscovensis RT (GCA_016292235.1); *Ca*. Thiothrix singaporensis SSD2 (GCA_013693955.1); *Ca*. Thiothrix anitrata A52 (GCF_017901155.1); *Thiothrix* sp. 207 (GCA_018813855.1); MAG *Thiothrix* sp. GKL-02 (GCA_029972225.1); MAG *Thiotrichaceae* sp. GKL-01 (GCA_029972135.1). The red box indicates the new MAGs obtained in this work.

**Figure 4:**
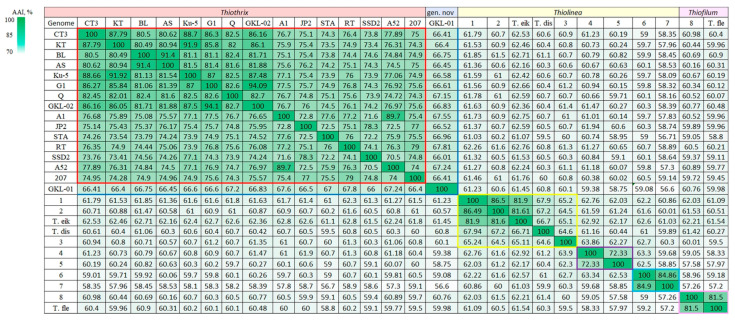
Heatmap of pairwise AAI values (%) for the assembled genomes of *Thiothrix*. *T*. *winogradskyi* CT3^T^ (GCA_021650935.1); ‘*Ca*. Thiothrix sulfatifontis’ KT (GCA_022828425.1); *T*. *lacustris* BL^T^ (GCF_000621325.1); *T*. *litoralis* AS^T^ (GCF_017901135.1); *T*. *subterranea* Ku-5^T^ (GCF_016772315.1); *T*. *caldifontis* G1^T^ (GCF_900107695.1); *Thiothrix* sp. STA_22 (GCA_028714775.1); *T*. *unzii* A1^T^ (GCA_017901175.1); *T*. *nivea* JP2^T^ (GCF_000260135.1); *T*. *fructosivorans* Q^T^ (GCA_017349355.1); *Ca*. Thiothrix moscovensis RT (GCA_016292235.1); *Ca*. Thiothrix singaporensis SSD2 (GCA_013693955.1); *Ca*. Thiothrix anitrata A52 (GCF_017901155.1); MAG *Thiothrix* sp. 207 (GCA_018813855.1); MAG *Thiothrix* sp. GKL-02 (GCA_029972225.1); *Thiotrichaceae* sp. GKL-01 (GCA_029972135.1); 1—uncultured *Thiothrix* sp. (GCA_937876535.1); 2—*Thiothrix* sp. Bin_56_1 (GCA_008015155.1); ‘*Thiolinea eikelboomii*’ AP3^T^ (GCA_900167255.1); *Thiolinea disciformis* B3-1^T^ (GCA_000371925.1); 3—Proteobacteria bacterium DOLZORAL124_45_7 (GCA_002747435.1); 4—Bacterium DOLJORAL78_51_81 (GCA 002746895.1); 5—*Thiotrichaceae* bacterium HLG_WM_MAG_08 (GCA_902727945.1); 6—*Thiothrix* sp. HKST-UBA238 (GCA_020442915.1); 7—*Thiothrix* sp. HKST-UBA237 (GCA_020442945.1); 8—*Thiofilum* sp. Hade_18-Q3-R52-61_MAXAC.390 (GCA_016711335.1); *Thiofilum flexile* EJ2M-B^T^ (GCA_000380185.1). Colored boxes indicate groups of phylogenetically close representatives.

A search against GTDB placed the bacterium GKL-01 in the family *Thiotrichaceae*, representing a novel genus. The average nucleotide identity (ANI) comparison values between MAG *Thiotrichaceae* sp. GKL-01 and other genomes of the genus *Thiothrix* accounted for 68.45–71.08%, which was lower than the threshold of 75% for the genera differentiation [18] (Figure 3). The average amino acid identity (AAI) comparison values between MAG GKL-01 and genomes of other members of the genus *Thiothrix* were in the range of 66.0–67.8%, which was slightly above the proposed threshold of 65% for the same genus [19] (Figure 4). MAG GKL-01 with AAI below 62% proved to be more distant from other *Thiotrichaceae* genera.

**Figure 5:**
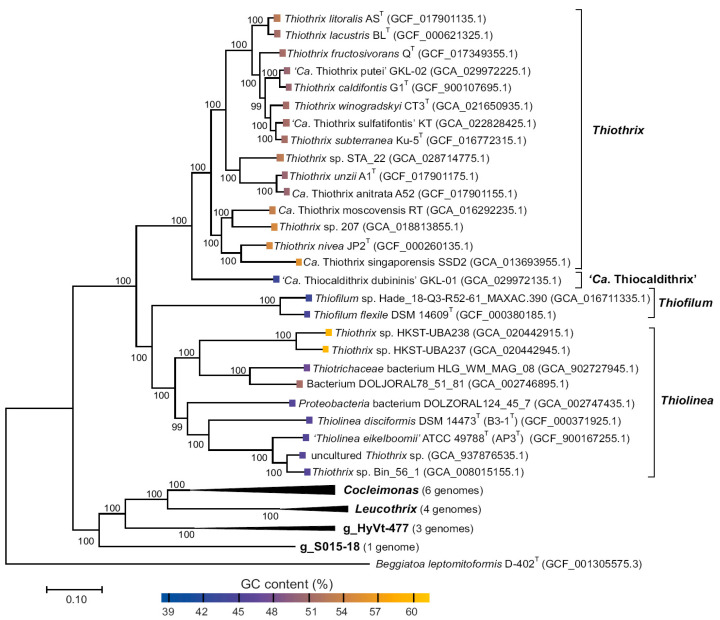
Genome-based phylogenetic tree of the family *Thiotrichaceae*. The genome positions were determined by the maximum likelihood method using concatenated sequences of 120 conserved marker genes. The GenBank assembly accession numbers are listed after the genome names. The genome of *Beggiatoa leptomitoformis* D-402^T^ (GCF_001305575.3) was used for tree rooting.

Phylogenetic position of MAG *Thiotrichaceae* sp. GKL-01, along with the previously described *Thiothrix* species, was analyzed by constructing a phylogenetic tree based on concatenated sequences of 120 conserved marker genes (Figure 5).

MAG *Thiotrichaceae* sp. GKL-01 formed a separate lineage at the root of the genus *Thiothrix*. Additionally, genome GC-content (44%), which meaningfully differs from the GC-content of other representatives of the genus *Thiothrix* (Figure 5), serves as a forcible argument for establishing the taxonomic position of MAG *Thiotrichaceae* sp. GKL-01 at the genus rank. Overall, the data obtained allowed us to identify MAG *Thiotrichaceae* sp. GKL-01 as a new species and genus within the family *Thiotrichaceae*, and we propose to name it ‘*Candidatus* Thiocaldithrix dubininis’ gen. nov., sp. nov. GKL-01.

It was previously shown that the classical phylogenetic marker 16S rRNA is sometimes not suitable for reliable determination of the taxonomy position of representatives within the genus *Thiothrix* because 16S rRNA homology accounts for 93.7–100% within the genus *Thiothrix*. Thus, the 16S rRNA data obtained for MAG *Thiothrix* sp. GKL-02, when compared with *Thiothrix* representatives (94.21–99.86%), do not allow unambiguously attribute it to one of *Thiothrix* species (Figure 3). However, the pairwise ANI and dDDH comparison values between MAG *Thiothrix* sp. GKL-02 and genomes of the earlier described species of the genus *Thiothrix* ranged in the intervals that lay between 74.6–93.2% and 22.7–57.3%, respectively (Figure 3 and Figure S1). The obtained distances between the genomes allowed us to assign GKL-02 to a new species of the genus *Thiothrix* and propose the name ‘*Candidatus* Thiothrix putei’ sp. nov. GKL-02. The branching topology of the tree based on 120 conserved protein genes suggests that *T*. *caldifontis* G1^T^ is the closest relative of GKL-02 (Figure 5).

The representatives of the genera *Thiolinea* and *Thiofilum*, phylogenetically and phenotypically related to the genus *Thiothrix*, belong to FCSB. Analysis of genome sequences that were attributed to the genus *Thiolinea* according to GTDB showed that AAI indexes (less than 65%) suggested dividing this genus into three genera (Figure 4): 1—genus *Thiolinea* inclusive uncultured *Thiothrix* sp. (GCA_937876535.1), *Thiothrix* sp. Bin_56_1 (GCA_008015155.1), ‘*Thiolinea eikelboomii*’ AP3^T^ (GCA_900167255.1), *Thiolinea disciformis* B3-1^T^ (GCA_000371925.1), Proteobacteria bacterium DOLZORAL124_45_7 (GCA_002747435.1); 2—a new putative genus inclusive Bacterium DOLJORAL78_51_81 (GCA 002746895.1) and *Thiotrichaceae* bacterium HLG_WM_MAG_08 (GCA_902727945.1); 3—a new putative genus inclusive *Thiothrix* sp. HKST-UBA238 (GCA_020442915.1) and *Thiothrix* sp. HKST-UBA237 (GCA_020442945.1).

### 2.3. Genome Analysis of ‘Ca. Thiothrix putei’ sp. nov. GKL-02 and ‘Ca. Thiocaldithrix dubininis’ gen. nov., sp. nov. GKL-01

Analysis of ‘*Ca*. Thiothrix putei’ sp. nov. GKL-02 genome showed the presence of a number of genes of dissimilatory sulfur metabolism, which is in accordance with the data for the members of filamentous colorless sulfur bacteria of the genus *Thiothrix* [20] (Table 2). Genomes of all members of the genus *Thiothrix*, including ‘*Ca*. Thiothrix putei’ GKL-02 contain genes of the branched SOX-system (*soxAXBYZ*) of oxidation thiosulfate to sulfur and sulfate; genes of the system of oxidation hydrogen sulfide to elemental sulfur *sqr*, sulfide:quinone oxidoreductase (*sqrF*, *sqrA*) and FCSD, flavocytochrome sulfide dehydrogenase (*fccAB*); genes of rDSR-complex (*dsrABEFHNEMKLJONR*) for oxidation sulfur to sulfite; genes of quinone-dependent sulfite dehydrogenase (*soeABC*) for direct oxidation of sulfite to sulfate and of ATP-sulfurylase, dissimilatory-type (*sat*), and APS-reductase (*aprAB*) for indirect oxidation.

High variability in the composition of nitrogen metabolism genes in members of the genus *Thiothrix* was previously determined in the gene sets of dissimilatory and assimilatory nitrate reduction as well as nitrogen fixation [6,7,20] (Table 3).

In the genome of ‘*Ca*. Thiothrix putei’ GKL-02, we have observed only the genes of the membrane-bound nitrate reductase *narGHI*, participating in the dissimilatory reduction of nitrogen, and genes of nitric oxide reductase *norBC*, catalyzing the reduction of NO to N_2_O that suggests the incomplete denitrification. Moreover, we have found in this genome the genes responsible for maturation of the nitrogenase complex (*nifASUVNWMT*), while genes encoding catalytic subunits have not been identified (Table 3).

Genes of assimilatory nitrate reduction (*nasA*, *nasD*) are absent from the genome of ‘*Ca*. Thiothrix putei’ GKL-02, similar to the genome of *Ca*. Thiothrix anitrata A52, but the classical amination genes (*glnB*, *gltBD*, *aspB*) were detected.

Similar to all members of the genus *Thiothrix*, the genome of ‘*Ca*. Thiothrix putei’ GKL-02 harbors genes for carbon dioxide autotrophic assimilation in the Calvin–Benson–Bassham cycle. Of RuBisCO types IAq, IAc, and II encountered in the genus [20], types IAq and IAc were found in the GKL-02 genome (Table 3).

Similar to the earlier described thirteen members of the genus *Thiothrix*, in ‘*Ca*. Thiothrix putei’ GKL-02, two phylogenetically distant copies of phosphoribulokinase were detected.

All genes of the Krebs cycle, oxidative pentose phosphate pathway, and glyoxylate shunt were found in the GKL-02 genome. The respiratory type of metabolism is determined by the presence of electron-transport chain genes (Table S1). Similar to that in all members of the genus *Thiothrix*, in the genome of GKL-02, the genes of FAD-dependent membrane-bound malate quinone oxidoreductase (*mqo*) are present instead of NAD-dependent malate dehydrogenase.

Phosphorus metabolism in ‘*Ca*. Thiothrix putei’ GKL-02 is presented in a similar way as in the previously described members of the genus *Thiothrix* by the systems of gene expression regulation (*phoURB*) of inorganic phosphorus transmembrane transport and its concentration (*pstSACB*), formation, and hydrolysis of phosphodiester bonds by polyphosphate kinase and exopolyphosphatase (*ppk1*, *epp*).

The genome of ‘*Ca*. Thiocaldithrix dubininis’ gen. nov., sp. nov. GKL-01 was analyzed in comparison with the previously described closely related genera, *Thiothrix*, *Thiolinea*, and *Thiofilum* (Table 2). The sulfur oxidation systems are the most conserved part of genomes in all four genera of the family *Thiotrichaceae*. The genes of oxidation of hydrogen sulfide to elemental sulfur (*sqrF*, *sqrA*, *fccAB*), elemental sulfur to sulfite (rDSR), direct oxidation of sulfite to sulfate (*soeABC*), as well as branched SOX-system (*soxAXBYZ*), were identified in all known members of the four genera, which argues for the close evolution in the systems with the high content of reduced sulfur compounds.

However, in contrast to the genus *Thiothrix*, ‘*Ca*. Thiocaldithrix dubininis’ GKL-01, similar to the representatives of the genera *Thiolinea* and *Thiofilum*, has no genes for indirect sulfite oxidation (*sat*, *aprAB*). Comparative analysis of nitrogen metabolism genes in ‘*Ca*. Thiocaldithrix dubininis’ GKL-01 and the closely related genus *Thiothrix* disclosed significant differences. Nitrogen metabolism in the GKL-01 bacterium is presented only by genes of classical amination (*glnB*, *gltBD*, *aspB*), whereas in representatives of the genus *Thiothrix*, there are genes of denitrification, assimilatory nitrate reduction, and nitrogen fixation. The profiles of nitrogen metabolism gene composition in ‘*Ca*. Thiocaldithrix dubininis’ GKL-01 and genera *Thiolinea* and *Thiofilum* are close, all lacking genes for denitrification and nitrogen fixation— the composition of RuBisCo types in ‘*Ca*. Thiocaldithrix dubininis’ GKL-01 (type II) is more close to genera *Thiolinea* and *Thiofilum*. It was found that the GKL-01 genome contains a catalase gene (*katE*) present in *Thiolinea* but absent in the representatives of genera *Thiothrix* and *Thiofilum*, but lacks *katG*, which is present in the representatives of *Thiolinea* and *Thiofilum*, and in most *Thiothrix* genomes.

### 2.4. Energy Converter Genes from the Phosphate Metabolism Group

The data presented in Table 3 and Table 4 show that members of the family *Thiotrichaceae* are characterized by a wide range of metabolic capabilities [4]. This raises interest in their energy conversion systems. To date, energy-converting mechanisms operating on both hydrogen and sodium ions are known in the bacterial world [21,22,23]. While H^+^-motive energy converters have been known since the publication of P. Mitchell’s theory [24], the first data on a wide variety of Na^+^-motive mechanisms, including Na^+^-decarboxylase [25], Na^+^-NADH-quinone oxidoreductase [26], Na^+^-ATPase [27], Na^+^-pyrophosphatase (PPase) [28], Na^+^-proteorhodopsin [29], and Na^+^-*cbb*_3_ cytochrome oxidase [30,31], appeared much later.

**Figure 6:**
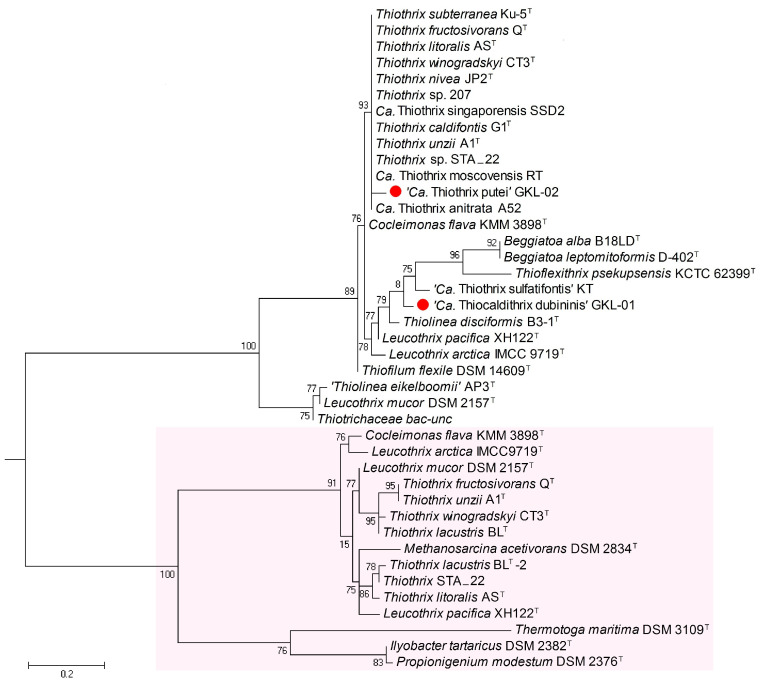
Maximum-likelihood tree based on the predicted amino acid sequences of F_0_F_1_-ATPase c-subunit mainly of the closest representatives of the family *Thiotrichaceae* and some other representatives of the order *Thiotrichales*. An unrooted tree with the highest log likelihood is shown. The bootstrap values (100 replicates) are shown next to the branches. The sequences were obtained from NCBI (https://blast.ncbi.nlm.nih.gov/Blast.cgi), RAST server (https://rast.nmpdr.org/rast.cgi) and UniProtKB (https://www.uniprot.org/uniprotkb/). Two large clusters presenting two ATPase subfamilies are denoted: the prospective Na^+^-ATPases (pink-colored rectangle at the bottom) and H^+^-motive F_0_F_1_ATPases (uncolored clusters). Amino acid sequences of c-subunits of *Propionigenium modestum*, *Ilyobacter tartaricus*, *Thermotoga maritima*, and *Methanosarcina acetivorans* were used as proven Na^+^-ATPases. The newly proposed species ‘*Ca*. Thiothrix putei’ GKL-02 and the new species of a new genus ‘*Ca*. Thiocaldithrix dubininis’ GKL-01 in the family *Thiotrichaceae* are indicated by small red balls. The bar shows the scale of branch length in the number of substitutions per site. The gene accession numbers are listed in Supplementary Materials (Text S1).

As to the present study, we have confined it to the search for energy converter genes from the phosphate metabolism group. We have found that genomes of a number of *Thiotrichaceae* family members bear not only genes of operon *atpIBEFHAGDC* coding H^+^-motive F-ATPases but also genes of another operon *atpDCQRBEFG* coding potential Na^+^-motive F-ATPases which were previously proposed to be termed N-ATPases and referred to the N-subfamily of F-ATPases [32]. These ATPases form a distinct branch reliably separated from the cluster of H^+^-motive F-ATPases (Figure 6).

The new MAGs, ‘*Ca*. Thiocaldithrix dubininis’ gen. nov., sp. nov. GKL-01, and ‘*Ca*. Thiothrix putei’ sp. nov. GKL-02 appeared to bear only genes of the H^+^-motive F-ATPase. In addition, according to the phylogeny based on the amino acid sequences of the c-subunit of F-ATPase, the two new species are in separate clusters (Figure 6) and are distantly related, which favors the results of the genome-based phylogeny (Figure 5). In the c-subunit of N-ATPases of the *Thiotrichaceae* members, we found the following potential ligands of Na^+^ ions: Glu-32, Met-63, Glu-65, Ser-66, and Tyr-70 (numbering in c-subunit of *Ilyobacter tartaricus* Na^+^-ATPase), which is close to consensus sequence with a set of Na^+^-ligands in well-known Na^+^-motive F-ATPases of *Ilyobacter tartaricus* [33,34], *Propionigenium modestum* [27,35] and *Thermotoga maritima* [34,36].

The N-ATPases of *Thiotrichaceae* representatives differ slightly from these F-ATPases in that the nonpolar Val-63 is replaced by nonpolar Met-63, and the polar Thr-67 by nonpolar Ile/Val-67. It should be noted here that Thr-67 apparently does not participate in the formation of the Na^+^-binding coordination sphere, and the above replacement is irrelevant. This follows from cryo-electron and atomic force microscopy as well as X-ray crystallography data, according to which the Na^+^-binding coordination sphere in the Na^+^-translocating membrane-bound c-subunit of the Na^+^-motive F-ATPase of *I. tartaricus* is formed by Gln-32, Val-63, Glu-65, Ser-66, and Tyr-70 [34,37]. Such a coordination sphere gives a total of six coordination bonds sufficient to bind Na^+^ ions. Moreover, the replacement of Val-63 by Met-63 evidently is not significant since, according to the X-ray crystallographic data, the ligand of the Na^+^ coordination sphere donated from Val-63 is the backbone carbonyl oxygen [34].

Notably, modeling of the ion-coordination structure of the c-subunits of ATP synthase from *Methanosarcina acetivorans* demonstrates that even the presence of only 3 instead of 4–5 typical polar amino acid residues characteristic of tight Na^+^-binding in the Na^+^-coordination sphere of the c-subunit in *I. tartaricus* and its homolog in the *P. modestum* enzyme [27,35], is sufficient to display the Na^+^-motive behavior of *M. acetivorans* ATPase [38]. Moreover, according to the free energy computation, the selectivity of c-subunit for Na^+^ over H^+^ in the range of ATPases of several microorganisms smoothly decreases in compliance with the decrease in the number of specific ligands in the potential Na^+^-coordination sphere [38]. Thus, it can be assumed that a part of representatives of the family *Thiotrichaceae*, which forms a cluster, reliably separated on the phylogenetic tree from owners of H^+^-motive F-ATPases (marked in pink, Figure 6), possess Na^+^-motive F-ATPases.

**Figure 7:**
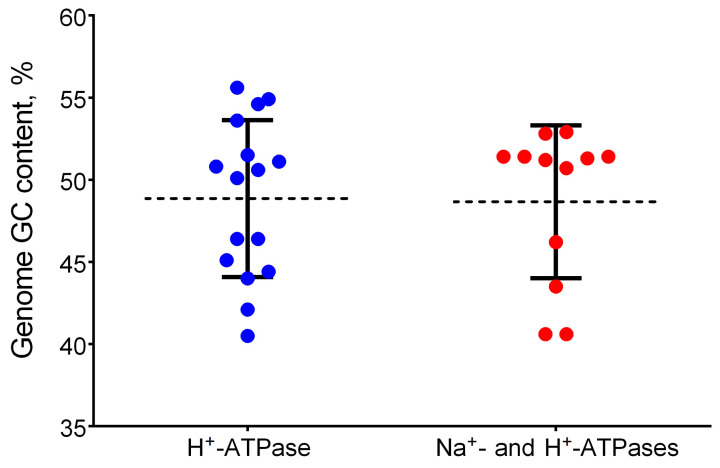
Genomic GC distribution depending on the presence or absence of Na^+^-ATPase genes in species of the order *Thiotrichales*. The mode of repeated measures one-way ANOVA data was used. Closed circles show genomic GC content of the only H^+^-ATPase-containing species (blue) and the species containing both H^+^- and Na^+^-ATPases (red); mean (dashed line, black) with SD (bars, black).

The reasons for the appearance of duplicate ATPases with different ion specificity in the genomes of a number of bacteria are not entirely clear. For a number of bacteria, it has been shown that GC content can reflect the adaptation of a species to certain environmental conditions; for example, an increase in GC correlates with an increase in the thermostability of bacteria [39]. Thus, the GC content could serve as a marker of the conditions and adaptive capabilities of a species. With the availability of a large number of genomes in the family *Thiotrichaceae*, an opportunity has arisen to use the GC index to test the correlation between the appearance of Na^+^-ATPases and the content of GC in the genome. As seen from Figure 7, the mean value of GC is the same in species that have only H^+^-motive F-ATPase genes and in species that have in their genomes both types of F-ATPases, H^+^-motive and Na^+^-motive.

These data show the lack of correlation between the appearance of Na^+^-ATPases and GC content in the genome and mediate the probable absence of influence of factors correlating with changes in the GC content. Moreover, the presence or absence of genes of dissimilatory nitrate and thiosulfate reduction are not related to the appearance of sodium-type energy converters (Table 4). Interestingly, according to Dibrova et al. [32], there is a correlation between the GC content of N-ATPase operons and the genomes of their hosts in representatives of different orders. If we take into account the assumption of these authors about the spread of N-ATPase between organisms by horizontal transfer of the operon, then it is very likely that it must have taken quite a long time to match the GC profile of the insertions to the GC profile of the host. If these assumptions are correct, then N-ATPases may be a very ancient acquisition of organisms.

**Figure 8:**
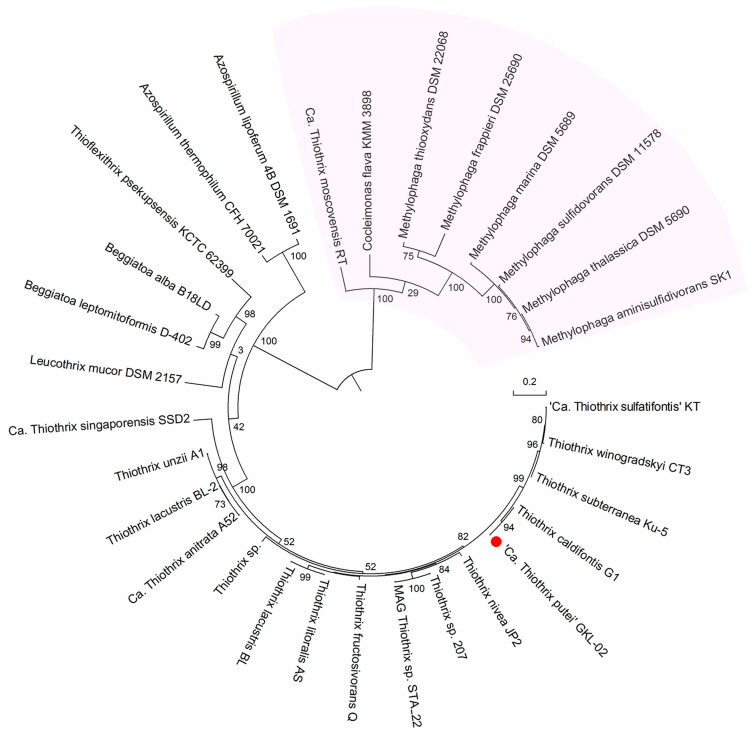
Maximum-likelihood tree based on the predicted amino acid sequences of membrane-bound PPases (*hppA*) of close members of the order *Thiotrichales* and two species of *Azospirillum*. The bootstrap values (100 replicates) are shown next to the branches of the unrooted tree. The sequences were obtained from NCBI (https://blast.ncbi.nlm.nih.gov/Blast.cgi), RAST server (https://rast.nmpdr.org/rast.cgi) and UniProtKB (https://www.uniprot.org/uniprotkb/). Two large clusters present two membrane-bound PPase subfamilies: Na^+^-PPases (pink-colored sector) and H^+^-PPases (uncolored sector). The newly proposed species ‘*Ca*. Thiothrix putei’ GKL-02 in the genus *Thiothrix* is indicated by a small red ball. The bar shows the scale of branch length in the number of substitutions per site. The gene accession numbers are listed in the Supplementary Materials (Text S2).

We found that a number of *Thiotrichales* species have genes not only for Na^+^-dependent F-ATPase but also genes for other energy converters of the phosphate metabolism group, namely, genes for Na^+^-translocating membrane PPases. The membrane PPases we consider here, unlike membrane ATPases, are encoded by only one *hppA* gene and are capable of energizing membranes during pyrophosphate hydrolysis. Similar to membrane ATPases, these PPases are electrogenic reversible enzymes. In the genomes of most members of the family *Thiotrichaceae*, including the newly proposed species ‘*Ca*. Thiothrix putei’ GKL-02, but not in ‘*Ca*. Thiocaldithrix dubininis’ GKL-01, we found the *hppA* gene encoding a membrane K^+^-independent H^+^-motive PPase (Figure 8).

In contrast to K^+^-independent PPase, membrane-bound K^+^-dependent PPase, which is encoded by the same gene with a single specific substitution, is a Na^+^-pump. Such a membrane-bound K^+^-dependent Na^+^-motive PPase, similar to that demonstrated in anaerobic archaea [28,40], is found in the genomes of some representatives of *Thiotrichaceae*, including *Ca*. Thiothrix moscovensis RT. Interestingly, in the *Thiotrichaceae* species that we analyzed, we did not detect the co-presence of PPase genes with different ionic specificity similar to what we observed with F-ATPase genes. It is believed that ion-translocating PPases maintain the potential across the membrane when ATP deficiency occurs in the cell. Thus, it can be assumed that representatives of *Thiotrichaceae* are ready to reflect unfavorable circumstances.

It would be tempting to assume that potential Na^+^-motive ATPases detected in representatives of the order *Thiotrichales* may have a physiological function of ATP synthesis, i.e., perform the role of ATP synthetase. In this case, the reason for the duplication of the ATPase function and conservation of the Na^+^-specific ATPases in them by natural selection with the simultaneous presence of H^+^-motive ATPases could be explained. Since many representatives of the order *Thiotrichales* do not belong to marine species and do not live in environments with high salt content, it seems unlikely that Na^+^-motive ATPases perform exclusively the function of sodium pumping from the cell. It seems to us that the presence of Na^+^-specific ATPases is rather related to the ability of many species of this group to inhabit niches with microoxic and maybe even anaerobic conditions.

During the transition to these conditions from normoxic conditions, the energy efficiency of the oxygen electron transport chain (ETC) decreases because the redox potential difference from the beginning of the ETC to its terminal site decreases. Under these conditions, the transition to sodium energetics would mean the transition to a more economical energy regime due to the fact that biomembranes have less leakage by sodium than by proton and consequently keep the electrochemical gradient of Na^+^ ions on the membrane longer. Then, under conditions of oxygen absence or deficiency, energy storage by oxidative phosphorylation involving Na^+^-motive ATP-synthetases would be more progressive than switching to substrate phosphorylation. At the same time, membrane PPases with both H^+^ and Na^+^ ion translocation functions could provide an additional safety net against a critical drop in the electrochemical gradient of H^+^ or Na^+^ ion concentration across the membrane.

### 2.5. Description of New Genus and Species

#### 2.5.1. Description of ‘Candidatus Thiocaldithrix’ gen. nov.

*Thiocaldithrix* (Thi.o.cal.di’thrix. Gr. neut. n. *theion* sulfur, brimstone, L. transliteration *thium*, sulfur; L. fem. adj. *caldus* hot; Gr. fem. n. *thrix* thread; N.L. fem. n. *Thiocaldithrix* is a filament existing in thermal spring in the presence of hydrogen sulfide).

#### 2.5.2. Description of ‘Candidatus Thiocaldithrix dubininis’ sp. nov.

- *dubininis* (du.bi’ni.nis. N.L. fem. gen. n. *dubininis* in honor of the Russian microbiologist Galina Dubinina, who made a significant contribution to the study of filamentous colorless sulfur bacteria).
- Not cultivated. Cells presumably perform a respiratory type of metabolism, facultative anaerobes, and lithoautotrophs. During lithoautotrophic growth, they obtain energy by oxidation of reduced sulfur compounds and carbon fixation in the Calvin–Benson–Bassham cycle. Potentially capable of anaerobic respiration in the presence of thiosulfate with the production of sulfide and sulfite due to thiosulfate reductase activity. Unable to participate in dissimilatory and assimilatory nitrate reduction and molecular nitrogen fixation. The 16S rRNA gene sequence is 94.7% identical to that of *T. nivea* JP2^T^.
- Source: MAG GKL-01 was obtained from the bacterial fouling metagenome of a hydrogen sulfide thermal spring.
- GC fraction of genomic DNA (%): 44 (genome sequence).
- GenBank accession number (whole genome assembly): GCA_029972225.1.

#### 2.5.3. Description of ‘Candidatus Thiothrix putei’ sp. nov.

- *putei* (pu.te’i. L. gen. n. *putei* of a well, from which the sample was taken, used to obtain this genome).
- Not cultivated. Cells presumably perform a respiratory type of metabolism, facultative anaerobes, and lithoautotrophs. During lithoautotrophic growth, they obtain energy by oxidation of reduced sulfur compounds and carbon fixation in the Calvin–Benson–Bassham cycle. Unable to participate in assimilatory nitrate reduction and molecular nitrogen fixation. Capable of dissimilatory anaerobic reduction of nitrate.
- Source: MAG GKL-02 was obtained from the bacterial fouling metagenome of a hydrogen sulfide thermal spring.
- GC fraction of genomic DNA (%): 50.8 (genome sequence).
- GenBank accession number (whole genome assembly): GCA_029972135.1.

## 3. Materials and Methods

### 3.1. Geography and Physicochemical Characteristics of Environmental Sampling Sites for Metagenomic Characterization of Thiocaldithrix sp. GKL-01 and Thiothrix sp. GKL-02

Biomass sampling of microbial fouling for metagenome analysis was performed in the sulfide hydrogen thermal spring in the town of Goryachiy Klyuch, Krasnodar region, Russia. The total community DNA was isolated from 500 mg of a microbial mat using a DNeasy PowerSoil DNA isolation kit (Qiagen, Hilden, Germany).

The physicochemical parameters of the water (pH, temperature, and redox potential) were measured with a HI18314F pH meter (Hanna Instruments, Vöhringen, Germany). The concentration of acid-labile sulfide in the samples was determined using the spectrophotometric method with *para*-phenylenediamine and by direct iodometric titration, preliminarily fixing the sulfide with 10% zinc acetate. The concentration of dissolved oxygen in the medium was determined using a HI 9142 oxygen meter (Romania). The total mineralization was determined by the method of electrical conductivity using a Multitest KSL-101 conductometer.

### 3.2. 16S rRNA Gene Profiling

Overall, 16S rRNA gene fragments were amplified from metagenomic DNA sample by PCR with the universal primers 341F (50-CCTAYGGGDBGCWSCAG) and 806R (50-GGACTACNVGGGTHTCTAAT) [41]. The obtained PCR products were bar-coded using the Nextera XT Index Kit v. 2 (Illumina, San Diego, CA, USA) and sequenced on an Illumina MiSeq instrument in a paired reads mode (2 × 300 nt). Pairwise reads were merged using FLASH v.1.2.11 [42]. Obtained 16S rRNA gene sequences were filtered to exclude low-quality and chimeric sequences and clustered into operational taxonomic units (OTUs) at a 97% identity threshold using the USEARCH v. 11 program [43]. To calculate the OTU abundances, the obtained reads were mapped to OTU sequences at a 97% global identity threshold by USEARCH. The OTUs composed of only a single read were discarded. The taxonomic assignment of the OTUs was performed using the VSEARCH v. 2.14.1 program and SILVA v.138 rRNA reference database [44].

### 3.3. Metagenome-Sequencing and Assembly of GKL-01 and GKL-02 MAGs

Metagenomic DNA isolated from Goryachiy Klyuch was sequenced using Illumina and Oxford Nanopore techniques. Sequencing on a MinION device (Oxford Nanopore Technologies, Oxford, UK) was performed using the ligation sequencing kit 1D and FLOMIN110 cells. A total of 3,084,557 reads containing 10.2 Gb (N50 of 9695 nt) were generated. The library for Illumina sequencing was prepared using the NEBNext Ultra II DNA library preparation kit (New England Biolabs, Ipswich, MA, USA). Sequencing of this library on an Illumina HiSeq2500 in a paired-end format (2 × 150 nt) produced 172.847.806 read pairs (about 52 Gb). Adapter sequences were removed with Cutadapt v.3.4 [45], and low-quality ends (q = 30) were trimmed using Sickle v.1.33 (https://github.com/najoshi/sickle, accessed on 24 July 2023).

MinION reads were assembled into contigs using Flye v. 2.8.2 in metagenome mode [46]. Several contig assemblies were generated using different parts of the total number of obtained MinION reads (from 10% to 100%, in 10% increments). The sequences of the assembled contigs were corrected using Illumina reads with two iterations of NextPolish v.1.4.1 [47].

The obtained contigs were binned into MAGs using MetaBAT v.2.15 [48]. The obtained MAGs were taxonomically classified using the Genome Taxonomy Database Toolkit (GTDB-Tk) v.1.5.0 [49] and the Genome Taxonomy database (GTDB) [3]. Two MAGs, obtained as circular contigs upon assembly of 20% and 30% of all MinION reads (designated GKL-02 and GKL-01, respectively), were assigned to the family *Thiotrichaceae*.

CheckM2 v.1.0.1 [50] was used to evaluate the completeness and contamination values of obtained MAGs.

### 3.4. Genome Analysis and Annotation

Gene search and annotation were carried out using the RAST server 2 [51], followed by manual correction of the annotation by comparing the predicted protein sequences with the National Center for Biotechnology Information (NCBI) databases. ANI was calculated using an online resource (https://www.ezbiocloud.net/tools/ani (accessed on 28 April 2023)) based on the OrthoANI algorithm, using USEARCH [52]. AAI between the genomes was determined using the aai.rb script from the enveomics collection [53]. dDDH calculation was performed using the GGDC online platform (https://ggdc.dsmz.de/ggdc.php# (accessed on 6 May 2023)).

For genome-based phylogenetic analysis, GTDB-Tk v.1.5.0 [49] was used to identify 120 single-copy marker genes in the genomes and to create multiple sequence alignments of concatenated amino acid sequences. The maximum likelihood tree was estimated from the alignment by PhyML v. 3.3 [54] using default parameters (LG amino acid substitution model, 4 substitution rates categories modeled by discrete gamma distribution with estimated shape parameter, branch support values calculated by approximate Bayes method).

### 3.5. Phylogenetic Analysis of F_0_F_1_-ATPase Subunits and Membrane-Bound PPases

Translated protein-coding genes were retrieved from the UniProtKB, NCBI, and RAST Server (version 2.0) databases. Protein-based phylogenetic analyses were fulfilled at the Phylogeny website (http://www.phylogeny.fr/, accessed on 8 June 2023) using Phylogeny Workspace Phylogenetic Analysis instruments as follows. The obtained amino acid sequences were aligned using MUSCLE software for multiple sequence alignment [55] and the Gblocks program to eliminate poorly aligned positions and divergent regions [56]. Phylogeny was estimated using the maximum likelihood method [57,58]. Final dendrograms were prepared using the MEGA 4.1 (beta) software [59].

## 4. Conclusions

Metagenomic analysis of a bacterial mat developing in a sulfidic thermal spring revealed two new candidate species of the family *Thiotrichaceae*, ‘*Candidatus* Thiothrix putei’ sp. nov. GKL-02 and ‘*Candidatus* Thiocaldithrix dubininis’ gen. nov., sp. nov. GKL-01, the latter representing a novel genus. Comparative genomic analysis showed that members of the family *Thiotrichaceae* have versatile metabolism, which indicates broad adaptive capabilities. An intriguing result was revealing features of two types of energetics in many members of *Thiotrichaceae*, which dispose of Na^+^-motive ATPases in addition to more common H^+^-motive ATPases. Another identified enzyme possessing Na^+^- and H^+^-motive forms was the less common membrane-bound PPase. The K^+^-independent and K^+^-dependent forms of this PPase, respectively, are capable of converting the energy of the proton and sodium ion gradient on the membrane into the high-energy bond of pyrophosphate and vice versa. Sodium-transporting forms of the above enzymes were previously described in anaerobic organisms. The reasons for the existence of such enzymes in facultatively aerobic members of *Thiotrichaceae* are not completely clear, but it can be assumed that their presence is associated with the functioning of the sodium cycle.

## Figures and Tables

**Table 1:** The general properties of genomes obtained from the metagenome of the microbial community of a sulfide thermal spring.

Genome (MAG)	Genome Assembly	Genome Size (MB)	GC-Content (%)	Genes		
				Protein-Coding	16S rRNA	tRNA
*Thiothrix* sp. GKL-02	GCA_029972225.1	4.28	50.8	4134	3	46
*Thiotrichaceae* sp. GKL-01	GCA_029972135.1	3.25	44.0	3021	3	49

**Table 2:** Characterization of the main metabolic pathways of the genera of the family *Thiotrichaceae. sqr*, sulfide: quinone oxidoreductase; *fccAB*, flavocytochrome sulfide dehydrogenase; *soxAXBYZ*, the branched SOX-system; rDsr, complex for sulfur oxidation *dsrABEFHNEMKLJONR*; *soeABC*, quinone-dependent sulfite dehydrogenase; *aprAB*, adenosine 5′-phosphosulfate reductase; *sat*, ATP-sulfurylase, dissimilatory-type; *phsABC*—thiosulfate reductase; *narG*, membrane-bound nitrate reductase; *napAB*, periplasmic nitrate reductase; *nasA*, assimilatory nitrate reductase; *nasB*—assimilatory nitrite reductase; *nirS*—dissimilatory nitrite reductase; *nirBD*—assimilatory nitrite reductase; *norBC*, nitric oxide reductase; *nosZ*, nitrous oxide reductase; *nif* gene cluster, nitrogenase genes; *mqo*, malate quinone oxidoreductase; *prk*, phosphoribulokinase; *hypABCDEF*, hydrogenase; SOD2, superoxide dismutase [Fe]; SOD1, superoxide dismutase, Cu-Zn family; *katG*, catalase/peroxidase HPI; PRDX5, peroxiredoxin 5; *katE*, catalase; *ccp*, cytochrome c_551_ peroxidase.

Metabolism	Characteristics		*Thiothrix*	‘*Ca* Thiocaldithrix’	*Thiolinea*		*Thiofilum Flexile*
					*Thiolinea disciformis*	‘*Thiolinea eikelboomii*’	
Sulfur metabolism	Hydrogen sulfide oxidation systems		*sqrA*, *sqrF*, *fccAB*	*sqrA*, *sqrF*, *fccAB*	*sqrA*, *sqrF*, *fccAB*		*sqrA*, *sqrF*, *fccA*
	SOX-system		*soxAXBYZ*	*soxAXBYZ*	*soxAXBYZ*		*soxAXBYZ*
	Elemental sulfur oxidation systems-rDSR		*dsrABEFHNEM* *KLJONR*	*dsrABEFHNEM* *KLJONR*	*dsrABEFHNEM* *KLJONR*		*dsrABEFHNEM* *KLJONR*
	Sulfite oxidation systems	Direct way	*soeABC*	*soeABC*	*soeABC*		*soeABC*
		Indirect way	*sat*, *aprAB*	−	−		−
	Thiosulfate Reductase		*phsABC*	*phsABC*	*phsABC*	−	−
Nitrogen metabolism	Dissimilatory nitrate reduction	NO_3_^−^ → NO^−^_2_	*narGHI*, *napAB* **	−	−	*narGHI*	*narGI*
		NO_2_^−^ → NH_3_	*nirBD* **	−	−		*nirBD*
		NO_2_^−^ → NO	*nirS* **	−	−		−
		NO → N_2_O	*norBC* **	−	−		−
		N_2_O → N_2_	*nosZ* **	−	−		−
	Nitrogen fixation		*nifASUBNXX2YB* *2ENQVWMHDKZTO* **	−	−		−
	Assimilatory nitrate reduction		*nasA*, *nasD* **	−	−		*nasA*, *nasD*
Carbon metabolism	Type of malate dehydrogenase		*mqo*	*mqo*	*mqo*		*mqo*, *mdh*
	Krebs cycle		+	+	+		+
	Glyoxylate pathway		+	+	+		+
	Pentose-phosphate pathway		+	+	+		+
	Type of RuBisCo		IAq, IAc, II **	II	II	IAq	−
	The type of phosphoribulokinase		*prk1*, *prk2*	*prk1*, *prk2*	*prk1*, *prk2*		*prk1*
	Hydrogenase		*hypABCDEF*	*hypABCDEF*	*hypABCDEF*		*hypABCDEF*
AOS-system	*sod2*		+	+	+		+
	*sod1*		−	−	+		−
	*prdx5*		+	*+*	*+*		−
	*katG*		+/− *	−	+		+
	*katE*		−	*+*	*+*		−
	*ccp*		+	+	+		+
	*gpx*		+	+	+		−

*—*katG* is present in the genomes of all *Thiothrix* except for *T*. *unzii* A1^T^, *Ca*. Thiothrix moscovensis RT and *Thiothrix* sp. 207. **—present in some species (see Table 3). The plus “+” and minus “−” symbols indicate presence or absence of a given gene(s).

**Table 3:** Distribution of genes for nitrogen metabolism and autotrophic carbon fixation in *Thiothrix* genomes. Genes: *narG*, membrane-bound nitrate reductase; *napAB*, periplasmic nitrate reductase; *nasA*, assimilatory nitrate reductase; *nasB*, assimilatory nitrite reductase; *nirS*, dissimilatory nitrite reductase; *nirBD*, assimilatory nitrite reductase; *norBC*, nitric oxide reductase; *nosZ*, nitrous oxide reductase; *nif* gene cluster, nitrogenase genes.

Strain	Type of RuBisCO	Dissimilatory Nitrate Reduction					Assimilatory Nitrate Reduction	N_2_ Fixation (*nif*)
		NO_3_^−^ → NO_2_^−^	NO_2_^−^ → NH_3_	NO_2_^−^ → NO	NO → N_2_O	N_2_O → N_2_		
*Thiothrix* sp. 207	IAq, IAc, II	*narGHI*, *napAB*	*nirBD*	*nirS*	*norBC*	*nosZ*	*nasA*, *nasD*	+
*Thiothrix* sp. STA_22	II	*narGHI*	*nirBD*	*nirS*	*norBC*	*nosZ*	*nasA*, *nasD*	+
*T*. *litoralis* AS^T^	IAq, IAc, II	*narGHI*	*nirBD*	*nirS*	*norBC*	−	*nasA*, *nasD*	+
*T*. *fructosivorans* Q^T^	IAq, II	*narGHI*	*nirBD*	*nirS*	*norBC*	−	*nasA*, *nasD*	−
*T*. *caldifontis* G1^T^	IAq, IAc, II	*narGHI*	*nirBD*	*nirS*	*norBC*	−	*nasA*, *nasD*	+
*T*. *winogradskyi* CT3^T^	IAc, II	*narGHI*	*nirBD*	*nirS*	*norBC*	−	*nasA*, *nasD*	−
*T*. *unzii* A1^T^	IAq, IAc, II	*narGHI*	*nirBD*	*nirS*	*norBC*	−	*nasA*, *nasD*	+
*Ca*. Thiothrix singaporensis SSD2	IAq, II	*narGHI*	*nirBD*	*nirS*	*norBC*	−	*nasA*, *nasD*	+
*Ca*. Thiothrix moscovensis RT	IAq, IAc, II	*narGHI*	*nirBD*	−	*norBC*	−	*nasA*, *nasD*	+
‘*Ca*. Thiothrix putei’ GKL-02	IAq, IAc	*narGHI*	−	−	*norBC*	−	−	−
*T*. *subterranea* Ku-5^T^	IAq, IAc, II	*narGHI*	*nirBD*	−	−	−	*nasA*, *nasD*	+
*T. lacustris* BL^T^	IAq, II	*narGHI*	*nirBD*	−	−	−	*nasA*, *nasD*	−
‘*Ca*. Thiothrix sulfatifontis’ KT	IAq, IAc, II	−	*nirBD*	−	−	−	*nasA*, *nasD*	−
*T*. *nivea* JP2^T^	IAq, IAc, II	*napAB*	*nirBD*	−	−	−	*nasA*, *nasD*	−
*Ca*. Thiothrix anitrata A52	IAq, IAc	−	−	−	−	−	−	−

**Table 4:** Interrelation of the physiological and ecological characteristics of the members of the family *Thiotrichaceae* and the presence of energy-coupling ATPase and membrane-bound PPase genes. Species bearing genes of Na^+^-pumps involved in phosphate metabolism are marked in pink color.

Genus	Strain	Biotope Conditions	Anaerobic Respiration		PPase, *hppA*		F_0_F_1_-ATPase	
			Thiosulfate Respiration (e^−^ Acceptor)	Dissimilatory Nitrate Reduction	Coupling Ion			
					Na^+^	H^+^	Na^+^	H^+^
*Thiothrix*	*Thiothrix* sp. STA_22	Unknown	−	+	−	+	+	+
	*T*. *litoralis* AS^T^	White Sea littoral, T 9–12 °C	+	+	−	+	+	+
	*T*. *fructosivorans* Q^T^	Activated sludge, T 4–28 °C	+	+	−	+	+	+
	*T*. *winogradskyi* CT3^T^	Activated sludge, T 20–24 °C	−	+	−	+	+	+
	*T*. *unzii* A1^T^	Active sludge of waste treatment facilities, T 20–28 °C	+	+	−	+	+	+
	*T. lacustris* BL^T^	Freshwater lake, T 9.3 °C	+	+	−	+	+	+
	*Ca*. Thiothrix moscovensis RT	Phosphorus removal bioreactor, T 20–24 °C	−	+	+	−	−	+
	*Thiothrix* sp. 207	Underground water, T 15 °C	+	+	−	+	−	+
	*T*. *caldifontis* G1^T^	Hydrogen sulfide thermal spring, T 35–40 °C	+	+	−	+	−	+
	*Ca*. Thiothrix singaporensis SSD2	Phosphorus removal bioreactor, T 20–24 °C	+	+	−	+	−	+
	‘*Ca*. Thiothrix putei’ GKL-02	Hydrogen sulfide thermal spring, T 35–40 °C	−	+	−	+	−	+
	*T*. *subterranea* Ku-5^T^	Spout from the shaft, T 15 °C	+	+	−	+	−	+
	‘*Ca*. Thiothrix sulfatifontis’ KT	Hydrogen sulfide spring, T 15–18 °C	+	−	−	+	−	+
	*T*. *nivea* JP2^T^	Sulfide-containing well water, T 16 °C	+	+ *	−	+	−	+
	*Ca*. Thiothrix anitrata A52	Hydrogen sulfide spring, T 18 °C	+	−	−	+	−	+
‘*Ca*. Thiocaldithrix’	‘*Ca*. Thiocaldithrix dubininis’ GKL-01	Hydrogen sulfide thermal spring, T 35–40 °C	+	−	−	−	−	+
*Thiolinea*	‘*Thiolinea eikelboomii*’ AP3^T^	Activated sludge, T 13–15 °C	−	+ * ȴ	−	−	−	+
	*Thiolinea disciformis* B3-1^T^	Activated sludge, T 14 °C	+	−	−	−	−	+
*Thiofilum*	*Thiofilum flexilie* EJ2M-B^T^	Activated sludge, T 10–14 °C	−	*−*	−	−	−	+
*Leucothrix*	*Leucothrix mucor* DSM 2157^T^	Shoreline of the harbor, T 28 °C	−	−	−	+	+	+
	*Leucothrix arctica* IMCC9719^T^	Arctic seawater, T 15 °C	−	−	−	−	+	+
	*Leucothrix pacifica* XH122^T^	Surface water, T 28 °C	−	−	−	−	+	+
*Cocleimonas*	*Cocleimonas flava* KMM 3898^T^	Internal tissue of a marine mollusk, T 25–30 °C	−	−	+	−	+	+
*Beggiatoa*	*Beggiatoa leptomitoformis* D-402^T^	Freshwater spring, T 8–35 °C	−	−	−	+	−	+
	*Beggiatoa alba* B18LD^T^	Rice paddy, T 0–38 °C	−	−	−	+	−	+

*—Despite the presence of relevant genes (Table 2), anaerobic growth has not been experimentally documented; ȴ—The genome cluster is incomplete and is represented only by *narGI*.

## Data Availability

Data sharing not applicable.

## Supplementary Materials

The following supporting information can be downloaded at https://www.mdpi.com/article/10.3390/ijms241814199/s1.
