# Supplementary material for: Metagenomics Revealed a New Genus ‘Candidatus Thiocaldithrix dubininis’ gen. nov., sp. nov. and a New Species ‘Candidatus Thiothrix putei’ sp. nov. in the Family Thiotrichaceae, Some Members of Which Have Traits of Both Na+- and H+-Motive Energetics
Source: Int J Mol Sci. 2023 Sep 17;24(18):14199. doi: 10.3390/ijms241814199 (PMC10532065; doi:10.3390/ijms241814199)
Supplement: Supplementary file 1 [file ijms-24-14199-s001.zip › ijms-2594872-supplementary.pdf]

# Supplementary Materials

# Metagenomics Revealed a New Genus 'Candidatus Thiocaldithrix dubininis' gen. nov., sp. nov. and a New Species 'Candidatus Thiothrix putei' sp. nov. in the Family Thiotrichaceae, Some Members of Which Have Traits of Both Na+- and H+-Motive Energetics †

**Nikolai V. Ravin 1,‡, Maria S. Muntyan 2,*,‡, Dmitry D. Smolyakov 3, Tatyana S. Rudenko 3, Alexey V. Beletsky 1,**
**Andrey V. Mardanov 1 and Margarita Yu. Grabovich 3,***

[1] Institute of Bioengineering, Research Center of Biotechnology, Russian Academy of Sciences, Leninsky Prospect, 33-2, 119071 Moscow, Russia; nravin@biengi.ac.ru (N.V.R.); mortu@yandex.ru (A.V.B.); mardanov@biengi.ac.ru (A.V.M.)
[2] Belozersky Institute of Physico-Chemical Biology, Lomonosov Moscow State University, Leninskie Gory, 119991 Moscow, Russia
[3] Department of Biochemistry and Cell Physiology, Voronezh State University, Universitetskaya pl., 1, 394018 Voronezh, Russia; songolifreya@mail.ru (D.D.S.); ipigun6292@gmail.com (T.S.R.)
* Correspondence: muntyan@genebee.msu.ru (M.S.M.); margarita_grabov@mail.ru (M.Y.G.)

† The study is dedicated to the memory of the outstanding bioenergeticist Prof. V.P. Skulachev.
‡ These authors contributed equally to this work.

**Table S1.** Characteristics of MAGs according to the minimal standards for description of MAGs (Bowers et. al.
*Nat. Biotechnol.* **2017**, *35*, 725–731. https://www.nature.com/articles/nbt.3893).

| *Thiotrichaceae* **sp. GKL-01** | |
|---|---|
| **General genome metadata** | |
| analysis project type | metagenome-assembled genome (MAG) |
| taxa id | multi marker approach |
| assembly software | Flye v. 2.9 |
| annotation | NCBI Prokaryotic Genome Annotation Pipeline (PGAP) |
| **Genome quality** | |
| assembly quaility | Finished: single 3,251,546 bp long contiguous sequence |
| completeness score | High Quality Draft: 100% |
| contamination score | High Quality Draft: 0.6% |
| completeness software | CheckM2 v. 1.0.1 |
| number of contigs | 1 |
| 16S recovered | yes |
| 16S recovery software | NCBI Prokaryotic Genome Annotation Pipeline (PGAP) |
| **MAG metadata** | |
| bin parameters | kmer+coverage |
| binning software | MetaBAT v.2.15 |

| *Thiothrix* sp. GKL-02 | |
|---|---|
| **General genome metadata** | |
| analysis project type | metagenome-assembled genome (MAG) |
| taxa id | multi marker approach |
| assembly software | Flye v. 2.9 |
| annotation | NCBI Prokaryotic Genome Annotation Pipeline (PGAP) |
| **Genome quality** | |
| assembly quaility | Finished: single 4,277,058 bp long contiguous sequence |
| completeness score | High Quality Draft: 100% |
| contamination score | High Quality Draft: 0.71% |
| completeness software | CheckM2 v. 1.0.1 |
| number of contigs | 1 |
| 16S recovered | yes |
| 16S recovery software | NCBI Prokaryotic Genome Annotation Pipeline (PGAP) |
| **MAG metadata** | |
| bin parameters | kmer+coverage |
| binning software | MetaBAT v.2.15 |

| dDDH | CT3 | KT | BL | AS | Ku-5 | G1 | STA | A1 | JP2 | Q | RT | SSD2 | A52 | 207 | GKL-02 | GKL-01 |
|---|---|---|---|---|---|---|---|---|---|---|---|---|---|---|---|---|
| CT3 | 100 | 33,5 | 27,9 | 27,6 | 40,1 | 36 | 22,6 | 35,1 | 23,7 | 32,6 | 30,5 | 22,3 | 38,9 | 21,5 | 29,7 | 13,8 |
| KT | 33,5 | 100 | 27,8 | 28,8 | 48,7 | 36,4 | 21,7 | 33,8 | 23,6 | 33,5 | 27,7 | 22,9 | 33,8 | 21,5 | 29,3 | 18,9 |
| BL | 27,9 | 27,8 | 100 | 56,4 | 27,7 | 28 | 26,2 | 27,8 | 23,9 | 28,8 | 24,9 | 22,4 | 27,7 | 26,4 | 28,2 | 20 |
| AS | 27,6 | 28,8 | 56,4 | 100 | 28,5 | 28,6 | 26,7 | 28,2 | 24,7 | 28,6 | 25,3 | 22,6 | 27,4 | 26,6 | 28,9 | 21,8 |
| Ku-5 | 40,1 | 48,7 | 27,7 | 28,5 | 100 | 38,5 | 27,3 | 36,7 | 23,6 | 34,7 | 27,9 | 22,4 | 35,9 | 27,3 | 40,4 | 21,8 |
| G1 | 36 | 36,4 | 28 | 28,6 | 38,5 | 100 | 27,3 | 37,3 | 23,8 | 32,9 | 30,2 | 22,7 | 35 | 28,4 | 57,3 | 19,7 |
| STA | 22,6 | 21,7 | 26,2 | 26,7 | 27,3 | 27,3 | 100 | 28,6 | 23,8 | 27,5 | 26 | 22,3 | 26,7 | 21,8 | 17,3 | 17,7 |
| A1 | 35,1 | 33,8 | 27,8 | 28,2 | 36,7 | 37,3 | 28,6 | 100 | 23,4 | 34,2 | 30,5 | 23 | 46,9 | 28,9 | 36 | 20 |
| JP2 | 23,7 | 23,6 | 23,9 | 24,7 | 23,6 | 23,8 | 23,8 | 23,4 | 100 | 23,7 | 25,6 | 27,3 | 24,6 | 28,4 | 26 | 19,7 |
| Q | 32,6 | 33,5 | 28,8 | 28,6 | 34,7 | 32,9 | 27,5 | 34,2 | 23,7 | 100 | 27,9 | 22,3 | 31,9 | 26,6 | 33,8 | 21,6 |
| RT | 30,5 | 27,7 | 24,9 | 25,3 | 27,9 | 30,2 | 26 | 30,5 | 25,6 | 27,9 | 100 | 24,9 | 21,3 | 27,4 | 28,5 | 24,3 |
| SSD2 | 22,3 | 22,9 | 22,4 | 22,6 | 22,4 | 22,7 | 22,3 | 23 | 27,3 | 22,3 | 24,9 | 100 | 32,3 | 25,6 | 22,7 | 21,4 |
| A52 | 38,9 | 33,8 | 27,7 | 27,4 | 35,9 | 35 | 26,7 | 46,9 | 24,6 | 31,9 | 21,3 | 32,3 | 100 | 27,6 | 36,3 | 22,1 |
| 207 | 21,5 | 21,5 | 26,4 | 26,6 | 27,3 | 28,4 | 21,8 | 28,9 | 28,4 | 26,6 | 25,6 | 25,6 | 27,6 | 100 | 21,5 | 16,6 |
| GKL-02 | 29,7 | 29,3 | 28,2 | 28,9 | 40,4 | 57,3 | 17,3 | 36 | 26 | 33,8 | 27,4 | 22,7 | 36,3 | 21,5 | 100 | 20,8 |
| GKL-01 | 13,8 | 18,9 | 20 | 21,8 | 21,8 | 19,7 | 17,7 | 20 | 19,7 | 21,6 | 21,4 | 21,4 | 22,1 | 16,6 | 20,8 | 100 |

**Figure S1.** Heatmap of pairwise dDDH values for the assembled genomes of *Thiothrix*. *T. winogradskyi* CT3$^T$ (GCA_021650935.1); '*Ca.* Thiothrix sulfatifontis' KT (GCA_022828425.1); *T. lacustris* BL$^T$ (GCF_000621325.1); *T. litoralis* AS$^T$ (GCF_017901135.1); *T. subterranea* Ku-5$^T$ (GCF_016772315.1); *T. caldifontis* G1$^T$ (GCF_900107695.1); *T. unzii* A1$^T$ (GCA_017901175.1); *T. nivea* JP2$^T$ (GCF_000260135.1); *T. fructosivorans* Q$^T$ (GCA_017349355.1); *Ca.* Thiothrix moscovensis RT (GCA_016292235.1); *Ca.* Thiothrix singaporensis SSD2 (GCA_013693955.1); *Ca.* Thiothrix anitrata A52 (GCF_017901155.1); *Thiothrix* sp. 207 (GCA_018813855.1); *Thiothrix* sp. STA_22 (GCA_028714775.1); *Thiotrichaceae* sp. GKL-01 (GCA_029972135.1); *Thiothrix* sp. GKL-02 (GCA_029972225.1).

## Text S1. Accession numbers of the subunit c gene of $F_0F_1$-ATPases

The gene accession numbers retrieved from UniProtKB and NCBI were as follows: *Thiothrix lacustris* BL - A0A1Y1QH02_9GAMM and A0A1Y1QMK8_9GAMM, *Thiothrix litoralis* AS - WP_210224368.1 and QTR46012.1, *Thiothrix winogradskyi* CT3 - WP_236501573.1 and UJS24521.1, *Thiothrix unzii* A1 - WP_210218492.1 and WP_210219344.1, *Thiothrix fructosivorans* Q - A0A8B0SKJ4_9GAMM and QTX11484.1, *Leucothrix arctica* IMCC 9719 - WP_109826210.1 and WP_109824171.1, *Leucothrix mucor* DSM 2834 - WP_022954344.1 and A0A7V2WW86_LEUMU, *Leucothrix pacifica* XH 122 - A0A317C0L7_9GAMM and A0A317CP54_9GAMM, *Cocleimonas flava* KMM 3898 - WP_131905866.1 and A0A4V2P7R0_9GAMM, *Thiolinea eikelboomii*-AP3 - A0A1T4XYD5_9GAMM, uncultured *Thiotrichaceae* bacterium - CAA6810787.1, *Ca.* Thiothrix anitrata A52 - QTR51456.1, '*Ca.* Thiothrix sulfatifontis' KT - UOG92218.1, *Thiothrix subterranea* Ku-5 - WP_202716014.1, '*Ca.* Thiotrichaceae bacterium' GKL-01 - WGZ91550.1, *Thiothrix caldifontis* G1 - WP_093066078.1, *Thiothrix nivea* JP2 - WP_002709454.1, *Ca.* Thiothrix singaporensis SSD2 - QLQ34252.1, *Beggiatoa leptomitoformis* D-402 - WP_201800170.1, *Beggiatoa alba* B18LD - I3CK18_9GAMM, *Thioflexithrix psekupsensis* KCTC 62399 - WP_086489165.1, *Thermotoga maritima* DSM 3109 - Q9X1V0|ATPL_THEMA, *Ilyobacter tartaricus* DSM 2382 - Q8KRV3|ATPL_ILYTA, *Propionigenium modestum* DSM 2376 - CAA37840.1. ATPase genes of the following species were retrieved from the corresponding genomes in the RAST database using BLAST: *Thiothrix* sp. GKL-02 ('*Ca* Thiothrix putei' GKL-02) - GCA_029972225.1, *Thiothrix* sp. STA_22 - GCA_028714775.1, *Thiofilum flexile* DSM 14609 – GCF_000380185.1, *Thiolinea disciformis* B31 - GCF_000371925.1, *Ca.* Thiothrix moscovensis RT - GCA_016292235.1, *Thiothrix* sp. SSD2 (*Ca*. Thiothrix singaporensis SSD2) - GCA_013693955.1, *Thiothrix* sp. 207 - GCA_018813855.1.

## Text S2. Accession numbers of the membrane PPase gene *hppA*

*Cocleimonas flava* KMM 3898 - WP_131904771.1, *Ca.* Thiothrix moscovensis RT - MBJ6611069.1, *Methylophaga thiooxydans* DSM 22068 - A0A0A0BCX4_9GAMM, *Methylophaga frappieri* DSM 25690 - WP_014705011.1, *Methylophaga marina* DSM 5689 - WP_286304297.1, *Methylophaga sulfidovorans* DSM 11578 - A0A1I3ZW22_9GAMM, *Methylophaga aminisulfidivorans* SK1 MP - F5T014_9GAMM, *Methylophaga thalassica* DSM 5690 **-** WP_284722875.1, *Beggiatoa leptomitoformis* D-402 - A0A2N9YBH2_9GAMM, *Beggiatoa alba* B18LD - WP_002682680.1, *Thioflexothrix psekupsensis* (*Thioflexithrix psekupsensis* KCTC 62399) - A0A251XAU4_9GAMM, *Thiothrix subterranea* Ku-5 - WP_202715708.1, *Thiothrix nivea* JP2 DSM 5205 - WP_002709561.1, *Thiothrix caldifontis* G1 - WP_093064126.1, *Ca*. Thiothrix singaporensis SSD2 - QLQ33052.1, *Thiothrix fructosivorans Q* - WP_207251832.1, *Thiothrix litoralis* AS - WP_210221415.1, *Thiothrix unzii* A1 - WP_210218856.1, *Ca.* Thiothrix anitrata A52 - WP_228292382.1, *Thiothrix winogradskyi* CT3 - WP_236500955.1, '*Ca.* Thiothrix sulfatifontis' KT - UOG93112.1, *Leucothrix mucor* DSM 2157 - A0A7V2T1F4_LEUMU, *Azospirillum lipoferum* DSM 1691 (strain 4B) - A0A5A9GUE1_AZOLI, *Azospirillum thermophilum* CFH 70021 - A0A2S2CQR0_9PROT. PPase genes (*hppA*) of the following species were retrieved from the corresponding genomes in the RAST database using BLAST: *Thiothrix* sp. GKL-02 ('*Ca* Thiothrix putei' GKL-02) - GCA_029972225.1, *Thiothrix* sp. 207 - GCA_018813855.1.
